# Efficiency, Kinetics and Mechanism of 4-Nitroaniline Removal from Aqueous Solutions by Emulsion Liquid Membranes Using Type 1 Facilitated Transport

**DOI:** 10.3390/membranes14010013

**Published:** 2024-01-01

**Authors:** Gerardo León, Asunción María Hidalgo, María Gómez, Elisa Gómez, Beatriz Miguel

**Affiliations:** 1 Departamento de Ingeniería Química y Ambiental, Universidad Politécnica de Cartagena, Paseo Alfonso XIII, 30203 Cartagena, Spain; beatriz.miguel@upct.es; 2 Departamento de Ingeniería Química, Universidad de Murcia, Campus de Espinardo, 30100 Murcia, Spain; ahidalgo@um.es (A.M.H.); maria.gomez@um.es (M.G.); egomez@um.es (E.G.)

**Keywords:** 4-nitroaniline, emulsion liquid membranes, efficiency, kinetics, transport mechanism

## Abstract

4-Nitroaniline (4NA) is a common organic pollutant that is released into the environment during the manufacture and processing of a wide variety of industrial products. This article describes the use of an emulsion liquid membrane process to remove 4NA from aqueous solutions using a type 1 facilitated transport mechanism. Optimization of the removal process was carried out by analyzing the efficiency of 4NA removal from the feed phase and the initial apparent feed/membrane fluxes and permeabilities under different experimental conditions. The kinetics of the removal process was analyzed using a simplified mass transfer model involving an empirical mass transfer coefficient calculated from experimental data, assuming that the concentrations of 4NA in the external aqueous phase and in the internal *w*/*o* emulsion are uniform. The results show that there is a very good fit between the experimental and model data and that the variation in the values of the overall mass transfer coefficients with the experimental conditions coincides with that of the removal efficiency mentioned above. The transport mechanism was studied by identifying the rate-controlling step of the removal process, using models described for adsorption processes, due to the strong parallelism between the transport mechanisms in adsorption and emulsion liquid membrane processes.

## 1. Introduction

The generation of potentially hazardous industrial effluents is an issue of growing concern today. Increasingly stringent environmental regulations require the development of technologies for the effective treatment of these industrial wastewaters containing hazardous pollutants. An investigation showed that 80% of streams in the USA contained organic pollutants [1]. Among these organic pollutants, nitroanilines constitute a group of compounds that have toxic effects on the environment and on human health [2].

4-Nitroaniline (4NA) is an aromatic amine widely used as an intermediate or precursor in the syntheses of azo dyes, pharmaceuticals, antioxidants, fuel additives, pesticides, corrosion inhibitors and oxidizing agents [2,3,4], being also present in some commonly used biosolid fertilizers [5,6]. Consequently, 4NA is discharged into the environment both directly in industrial effluents and through the application of such fertilizers, and indirectly, as a result of the degradation of some of the above mentioned compounds [7]. It is toxic through ingestion, inhalation, and contact with the skin, with a threshold limit value (TLV) of 0.001 kg/m^3^ [4]. Due to its non-biodegradability and environmental persistence, toxicity, carcinogenicity and mutagenesis, 4NA is considered as a toxic chemical by the USA Environmental Protection Agency and as a top-priority pollutant in China [8].

Therefore, the study of new technologies for the treatment of 4NA-contaminated water is a topic of great scientific interest. Several processes have been described for the removal of 4NA from water sources, including biodegradation [6,7,8,9], oxidation [10], reduction [11,12], adsorption [13,14,15], advanced oxidation processes [16,17,18,19] and pressure-driven membrane processes [20,21].

Liquid membrane separation processes have been applied as an effective tool for the removal of a wide variety of organic and inorganic compounds from aqueous solutions due to their ease of operation, high selectivity, combination of removal and recovery processes in a single step, low energy costs and simplicity of design [22]. A liquid membrane system consists of two miscible phases (feed and product phases) separated by a third immiscible phase (the membrane phase) [23]. According to their configuration, three groups of liquid membranes are usually considered: bulk, supported and emulsified. Emulsion liquid membranes are prepared by emulsifying the membrane and product phases and dispersing this emulsion in the feed phase, so that the membrane phase separates the product phase (encapsulated internal droplets) from the external feed phase [23].

To improve the effectiveness of the separation process, so-called facilitated transports are used to maximize the rate of extraction of the species to be separated and its release into the product phase, allowing the transport of the species against its concentration gradient [24]. In type 1 facilitation, a substance (stripping agent) is added to the product phase which reacts quantitatively with the diffusing species to produce a membrane-insoluble product, thereby reducing the concentration of that species to zero at the membrane/product interface and achieving a high concentration gradient of that species across the membrane phase [24].

**Figure 1:**
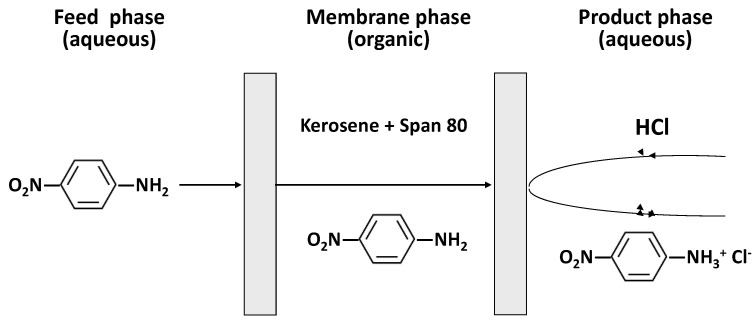
Type 1 facilitated transport of 4NA through emulsion liquid membranes.

This paper analyzes the efficiency, kinetics and mechanism of 4NA removal from aqueous solutions by emulsion liquid membranes using a type 1 facilitated transport mechanism occurring in a water-in-oil-in-water (*w*/*o*/*w*) emulsion consisting of an external feed aqueous phase containing 4NA, an internal aqueous product phase containing HCl as a stripping agent and a membrane phase composed of a solution of the surfactant Span 80 in kerosene as a solvent (Figure 1).

## 2. Materials and Methods

### 2.1. Reagents

Kerosene and sorbitan monooleate (Span 80) were supplied by Sigma Aldrich, Steinheim (Germany). 4-Nitroaniline (98%) and HCl (37%) were obtained from Panreac, Darmstadt (Germany). All chemicals were used without any further purification. Deionized water was used for making all the aqueous solutions.

### 2.2. Procedure

**Figure 2:**
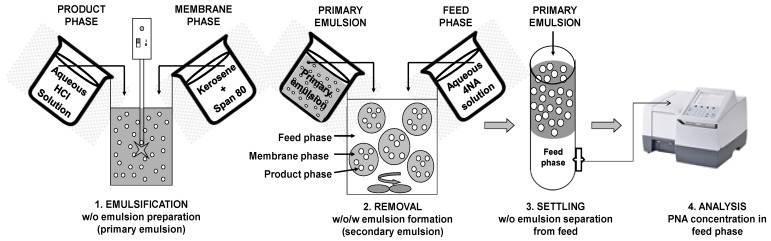
Schematic representation of steps involved in 4NA removal by emulsion liquid membranes.

The removal process involves four successive steps: 1—preparation of the primary water in oil emulsion, 2—removal of 4NA by contacting feed and primary emulsion to form the secondary emulsion (water-in-oil-in-water emulsion), 3—on samples taken for analysis, separation by decantation of the feed phase from the primary emulsion phase and 4—analysis of 4NA concentration in the feed phase to establish the efficiency of the removal process (Figure 2).

The feed phase was formed by 0.1 g/L aqueous solution of 4-nitroaniline, the membrane phase consisted of solutions of the surfactant Span 80 in kerosene, at concentrations ranging from 0.5% to 5.0%, and the product phase comprised aqueous solutions of hydrochloric acid ranging from 0.05 M to 0.50 M.

The primary water in oil emulsion (*w*/*o*) was prepared by mixing different volumes of the product phase (V_p_) and of the membrane phase (V_m_) by using a high-speed OMNI MIXER homogenizer (Omni International, Kennesaw, GA, USA), at 2700 rpm during 5 min. To prepare the water-in-oil-in-water emulsion (*w*/*o*/*w*) a volume of this primary emulsion (V_emul_) was then gradually added to a volume of the external feed phase (V_f_) in a glass cell equipped with a variable-speed propeller, stirring the mixture at a stirring rate ranging from 50 to 200 rpm. V_p_/V_m_ volume ratios ranging from 0.7 to 1.0 were analyzed at a constant V_f_/V_emul_ volume ratio of 2, while V_f_/V_emul_ volume ratios ranging from 1 to 8 were analyzed at a constant V_p_/V_m_ volume ratio of 1.

The duration of the experiments was 15 min, to ensure that in none of the studied experimental conditions there was a significant breakage of the emulsion globules; this should lead to an increase in the concentration of 4NA in the feed phase with time.

Samples of the secondary *w*/*o*/*w* emulsion were periodically taken and allowed to settle for 5 min to achieve separation of the feed phase and the primary *w*/*o* emulsion. A quantity of 1 mL of the feed phase was then analyzed, after the addition of 2 mL of 1 M HCl solution, by means of UV spectrophotometry at a wavelength of 243 nm using a UNICAM UV2 spectrophotometer (Unicam Limited, Cambridge, UK). The concentration of 4NA in the unknown sample was determined from the 4NA calibration curve. All the experiments were performed at room temperature and in duplicate. The results obtained showed a maximum deviation of 5%.

The typical experimental condition was: membrane phase 5% Span 80 in kerosene, product phase 0.5 M hydrochloric acid, stirring rate 200 rpm, V_f_/V_emul_ volume ratio 2 and V_p_/V_m_ volume ratio 1.

## 3. Results

### 3.1. Removal Efficiency

Removal percentage (RP), apparent initial flux (J) and apparent initial permeability (P) were used to study the efficiency of the 4NA removal process.

Percentage of 4NA removal from the feed phase (RP) was determined according to the Equation (1)

(1) $$\mathrm{RP}=\frac{C_{f,0}-C_{f,t}}{C_{f,0}}\times 100$$

where C_f,0_ and C_f,t_ are the initial and final (15 min) concentrations of 4NA in the external feed phase.

Initial apparent fluxes (J) and permeabilities (P) of 4NA through the feed/membrane interface were calculated from the slopes of the straight lines obtained when plotting, respectively, C_f_ and ln[C_f,t_/(C_f,0_] against time, during the first 3 minutes of the experiments, according to Equations (2) and (3) [25].

(2) $$J=-\frac{V_{f}\times {\mathrm{dC}}_{f}}{V_{\mathrm{emul}}\times \mathrm{dt}}$$

(3) $$\ln\frac{C_{f,t}}{C_{f,0}}=-\frac{V_{\mathrm{emul}}\times P\times t}{V_{f}}$$

where V_emul_ is the volume of the primary emulsion, internal phase volume plus membrane volume, V_f_ in the volume of the external feed phase and t is the contact time. These equations are used assuming that the membrane area is proportional to the emulsion volume, that the release reaction, which takes place at the membrane/product interface, is very fast preventing the accumulation of solute in the membrane phase and that there is uniformity in the size of the emulsion droplets when the membrane preparation conditions are the same [25].

**Figure 3:**
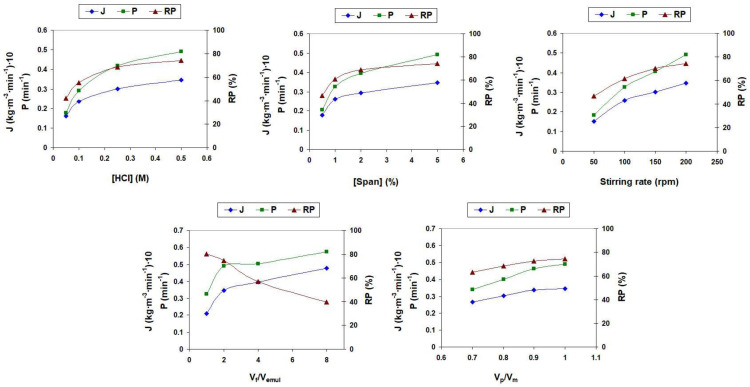
Removal percentage (RP), apparent initial flux (J) and apparent initial permeability (P) for 4NA removal by ELM under different experimental conditions.

Figure 3 shows the values of the 4NA removal percentage, apparent initial flux and apparent initial permeability under the different experimental conditions studied.

The increase in HCl concentration in the product phase from 0.05 M to 0.50 M leads to an increase in 4NA removal as a consequence of the increase in the stripping driving force, which favors the diffusion of 4NA from the feed/membrane interface to the membrane/product interface leading to an increase in 4NA transport from the feed to the membrane phase [26].

The 4NA removal increases as a surfactant concentration in the membrane phase increases from 0.5% to 5.0% due to the reduction in the interfacial tension between the phases resulting in smaller emulsion droplets that provide a larger mass transfer area, leading to an increase in 4NA transport [27].

The increase in the stirring speed from 50 to 200 rpm leads to an increase in 4NA removal due to the formation of smaller emulsion droplets, which provides a higher mass transfer area between the feed phase and the membrane phase, leading to an increase in the mass transfer rate [28].

The effect of the V_f_/V_emul_ ratio on the 4NA removal was studied at a constant V_p_/V_m_ ratio (V_p_/V_m_ = 1). Increasing the V_f_/V_emul_ ratio leads to an increase in the amount of 4NA that can be removed from the feed phase, but with no change in the amount of stripping agent in the product phase. This leads to an increase in the 4NA transport from the feed phase to the membrane phase that is manifested by an increase in flux and permeability (which is especially significant when the volume ratio increases from 1 to 2) and to a decrease in the removal percentage (the percentage of 4NA removed from the total 4NA present in the feed), which is especially significant at a volume ratio greater than 2, due to the significant increase in the number of 4NA molecules to be removed from the feed phase in comparison to the total number of 4NA molecules that can be effectively transported by a constant number of stripping agent molecules [29].

The effect of the V_p_/V_m_ ratio on 4NA elimination was analyzed at a constant V_f_/V_emul_ ratio (V_f_/V_emul_ = 2). The increase in the internal volume of the aqueous phase produces two opposite effects. On the one hand, it generates an increase in emulsion viscosity, which leads to an increase in the emulsion droplet size which decreases the mass transfer area and results in a decrease in removal efficiency [30]. On the other hand, it produces an increased ratio between the amount of stripping agent in the permeate phase and the amount of 4NA in the feed phase, which increases the stripping driving force, delaying the accumulation of 4NA in the membrane phase, and resulting in an increase in the removal efficiency [26]. The total result of these two effects is a slight increase in 4NP flux, permeability and removal efficiency.

### 3.2. Removal Process Kinetics

Among the different models that have been proposed to describe the kinetics of the transport process in emulsion liquid membranes [31,32,33,34], we selected in this study the model of Lin et al. [34] because of its simplicity and ease of calculation. This model assumes that the 4NA concentrations are uniform in both the external aqueous phase and the primary water in oil emulsion and is represented by Equation (4) [34], which is valid for times shorter than the final time.

(4) $$\ln\frac{C_{\mathrm{emul},\mathrm{tf}}}{C_{\mathrm{emul},\mathrm{tf}}-C_{\mathrm{emul},t}}=k\times t\;\;\;\;\;\;Y=k\times t$$

where C_emul,t_ and C_emul,tf_ are 4NA concentrations (mg/L) in the emulsion droplets at any time and at the final time (15 min), respectively, and k is the overall mass transfer coefficient (min^−1^).

4NA concentrations in the emulsion droplets (C_emul_) and in the external feed aqueous solution (C_f_) are related by the following material balance:

(5) $$V_{f}\times {(C}_{f,0}-C_{f,t})=V_{\mathrm{emul}}\times C_{\mathrm{emul},t}$$

where C_f,0_ and C_f,t_ are, respectively, the initial and time t concentrations of 4NA in the feed phase (mg/L), and V_f_ and V_emul_ are the volumes (mL) of the feed and the emulsion phases, respectively. 4NA concentrations (mg/L) in the emulsion droplets at the final time C_emul,tf_ were determined using Equation (5), C_f,t_ being the feed 4NA concentration at the final time (15 min).

**Figure 4:**
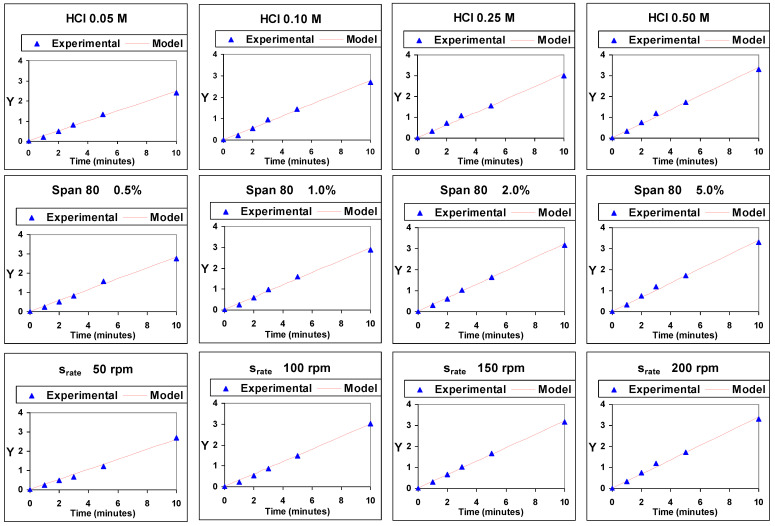

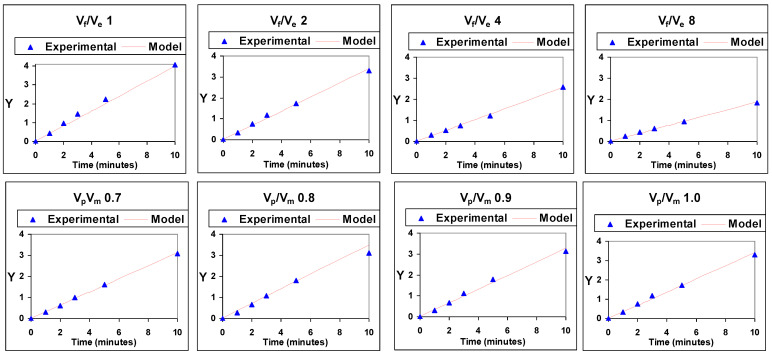
Representation of experimental and model values of ln[C_emul,tf_/(C_emul,tf_ − C_emul,t_)] (Y) against time.

Figure 4 shows the values of the representation against time of the experimental values of Y, ln[C_emul,tf_/(C_emul,tf_ − C_emul,t_)], and of those obtained by means of the model, under the different experimental conditions studied. A very good similarity between experimental and model values can be appreciated, which points to a good validity of the model.

**Figure 5:**
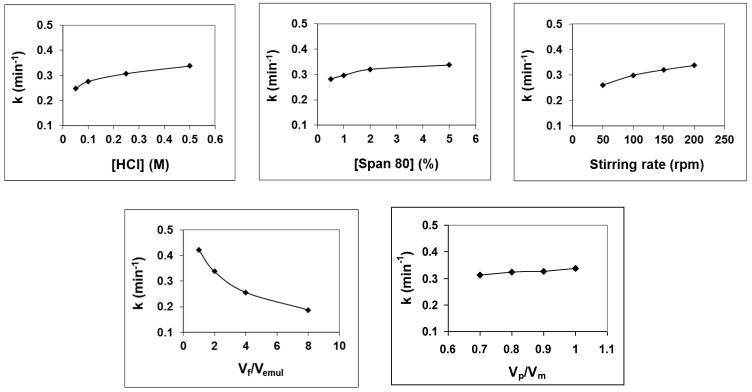
Overall mass transfer coefficients of 4NA removal under different studied experimental conditions.

In another way, Figure 5 shows the values of the overall mass transfer coefficient under those different experimental conditions. It can be observed that the variation in the overall mass transfer coefficients with the variation in different experimental conditions studied coincides with that of the 4NA removal efficiency analyzed above. That is, the increase in HCl concentration in the product, surfactant concentration in the membrane phase, stirring rate of the secondary *w*/*o*/*w* emulsion, and V_p_/V_m_ ratio lead to an increase in the value of the overall mass transfer coefficient, while the increase in the V_f_/V_emul_ leads to its decrease.

### 3.3. Transport Mechanism

**Figure 6:**
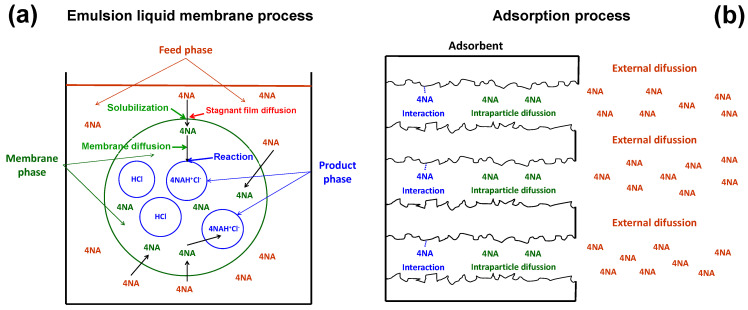
Transport mechanisms in emulsion liquid membrane (**a**) and adsorption processes (**b**).

The mechanism of 4NA transport through an emulsion liquid membrane by a type 1 facilitated transport includes four steps (Figure 6a) [31]:

1. 4NA diffusion through the stagnant film of the feed aqueous phase at feed/membrane interface.
2. 4NA solubilization into the membrane phase.
3. 4NA diffusion through the membrane phase to the membrane/product interface.
4. At the membrane/product interface, reaction of 4NA with the striping agent (HCl) present in the product phase to form a membrane phase insoluble product (4NAH^+^Cl^−^].

As the solubilization and reaction steps are much faster than those of the diffusion, the transport in a type 1-facilitated emulsion liquid membrane process will be governed by diffusion through the stagnant film of the feed aqueous phase at the membrane/phase interface or by diffusion through the membrane phase.

This elementary transport mechanism is very similar to that of the adsorption of an adsorbate (we will refer to the 4NA) onto an adsorbent, which includes three basic steps (Figure 6b) [35]:

1. External diffusion (film diffusion), transport of the adsorbate (4NA) from the bulk phase to the external surface of the adsorbent.
2. Intraparticle diffusion (pore diffusion), transport of the adsorbate (4NA) from the external surface into the pores.
3. Surface reaction, which is the attachment of the adsorbate to the internal surface of the adsorbent.

As indicated above, the reaction step is much faster than the diffusion steps, so transport in an adsorption process will be governed by either external diffusion or intraparticle diffusion.

This great parallelism between the mechanisms associated with the transport in both adsorption and type 1 emulsion liquid membrane processes makes it possible to assimilate the external diffusion and the intraparticle diffusion of the adsorption process with the diffusion through the stagnant film of the feed aqueous phase at the feed/membrane interface and the membrane–phase diffusion, respectively, of the emulsion liquid membrane process.

In order to establish the extent of an adsorption process, the parameter adsorption capacity is often used, which is usually defined, for both at any time t (q_t_) or at the final time or equilibrium (q_e_), as the amount of adsorbate retained per unit mass of adsorbent (mg/g) [35], according to Equations (6) and (7).

(6) $$q_{e}=\frac{{(C}_{0}-C_{e})\times V}{m}$$

(7) $$q_{t}=\frac{{(C}_{0}-C_{t})\times V}{m}$$

where C_0_, C_t_ and C_e_ were, respectively, the initial, time t and equilibrium adsorbate concentrations in the solution (mg/L), V the volume of the adsorbate solution (L) and m the mass of the adsorbent (g).

These parameters have been defined in the case of emulsion liquid membranes [36] as the amount of compound removed from the feed phase per volume unit of emulsion phase (mg/L), according to Equations (8) and (9).

(8) $$q_{e,\mathrm{ELM}}=\frac{{(C}_{f,0}-C_{f,e})\times V_{f}}{V_{\mathrm{emul}}}$$

(9) $$q_{t,\mathrm{ELM}}=\frac{{(C}_{f,0}-C_{f,t})\times V_{f}}{V_{\mathrm{emul}}}$$

where C_f,e_ is the equilibrium concentration (at 15 min) of 4NA in the feed phase (mg/L).

Accordingly, the amounts of 4NA removed from the feed phase per volume unit of the emulsion phase, at any time t (q_t_), and at 15 min (q_e_), were estimated from Equations (8) and (9).

Therefore, the mechanism of type 1-facilitated transport of 4NA through an emulsion liquid membrane was analyzed by means of two models developed for adsorption processes, the Weber and Morris intraparticle diffusion model and the Boyd model.

The Weber and Morris intraparticle diffusion model [37] is usually expressed according to Equation (10):

(10) $$q_{t}=k_{\mathrm{intp}}\times t^{1/2}+C_{i}$$

where k_intp_ (mg/g·h^1/2^) is the rate constant of the intraparticle diffusion (membrane diffusion in the case of ELM)) and Ci represents the effect of the external diffusion (diffusion through the stagnant film of the feed phase in the case of ELM). The value of these parameters is obtained from the slope and the intercept when q_t_ plotted against t^1/2^. If this representation is linear and passes through the origin, intraparticle diffusion is the only step that controls the rate of the process, but if the representation shows multilinearity, both external diffusion and intraparticle diffusion are involved in controlling the rate of the process.

When the latter occurs, the Boyd kinetic model [38] allows us to establish which of the two steps is the one that mainly controls the rate of the process. This model is described by the equation:

(11) $$B\times t=-0.4977-\ln\left(1-\frac{q_{t}}{q_{e}}\right)$$

If the plot of B·t versus time is a straight line passing through the origin, the transport process is mainly controlled by intraparticle diffusion; otherwise, it is mainly controlled by external diffusion.

**Figure 7:**
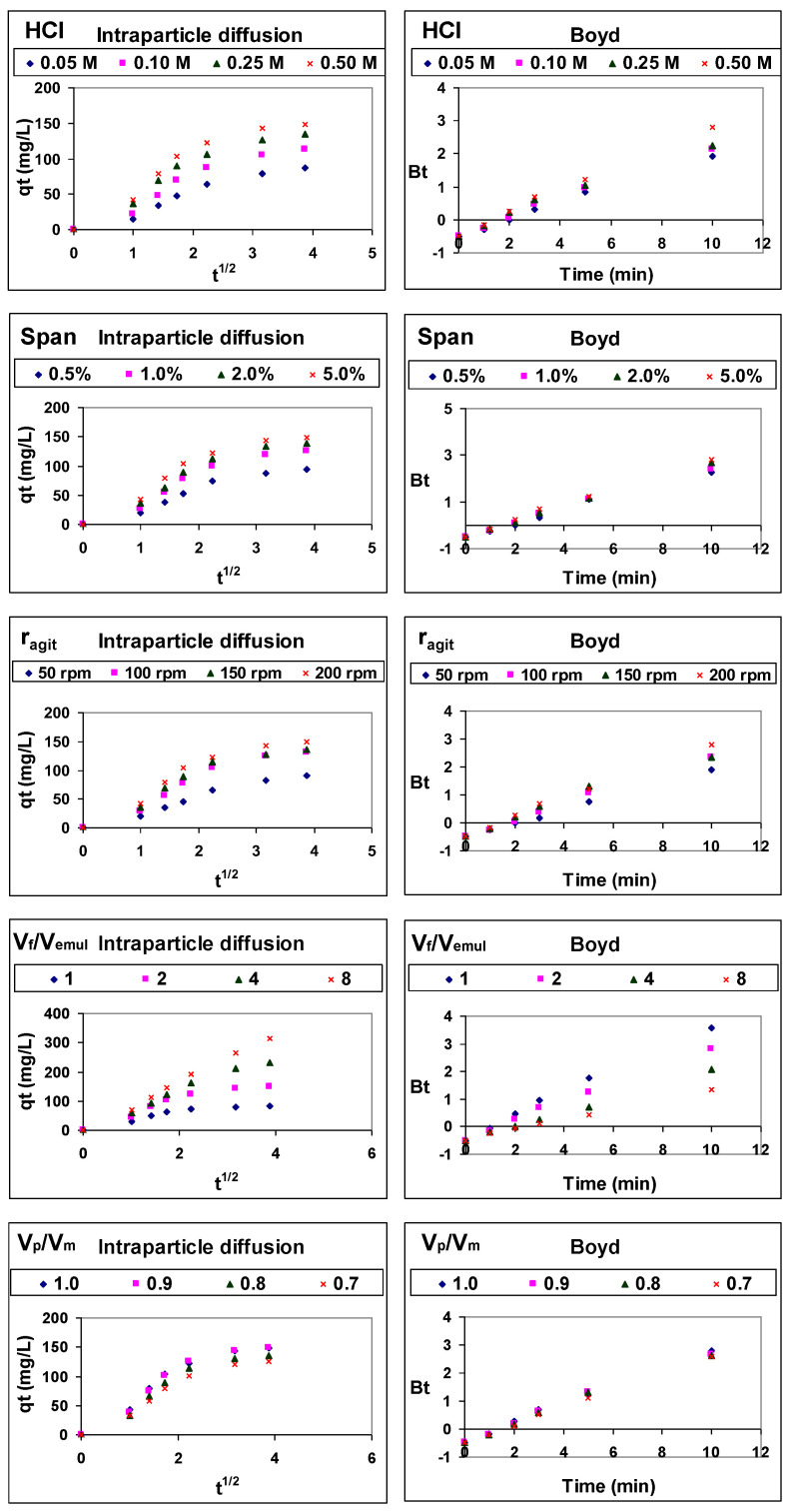
Weber–Morris and Boyd model representations of 4NA type 1-facilitated transport through ELM.

The results are presented in Figure 7. As this figure shows that Weber–Morris model representations are not linear over the entire time range, 4NA transport from the feed phase to the product phase is controlled by both the diffusion through the stagnant film of the feed aqueous phase at the feed/membrane interface and the diffusion through the membrane phase.

As Boyd model representations are not fully linear and they do not pass through the origin, it can be concluded that the rate of 4NA type 1-facilitated transport through emulsion liquid membranes is mainly controlled by 4NA diffusion through the stagnant film of the feed aqueous phase at the feed/membrane interface.

## 4. Conclusions

The removal of 4NA from aqueous solutions by emulsion liquid membranes using a type 1-facilitated transport mechanism was studied in this paper. In order to optimize the removal process, the efficiency of 4NA removal from the feed phase and the initial apparent feed/membrane fluxes and permeabilities were studied under different experimental conditions. The removal of 4NA increased by increasing the HCl concentration in the internal aqueous phase from 0.05 M to 0.50 M, by increasing the surfactant concentration in the membrane phase from 0.5% to 5.0%, by increasing the stirring speed from 50 to 200 rpm, by increasing the permeate/membrane volume ratio from 0.7 to 1, and by decreasing the feed/emulsion volume ratio from 8 to 1.

The kinetics of the removal process were analyzed using a simplified mass transfer model involving an empirical mass transfer coefficient calculated from experimental data. The results show a very good fit between the experimental and model data and that the values of the overall mass transfer coefficient with the experimental conditions coincide with those of the removal efficiency mentioned above.

Due to the great parallelism between the transport mechanisms of adsorption and emulsion liquid membrane processes, the mechanism of the type 1-facilitated transport of 4NA through ELM was studied by identifying the rate-controlling step of the process using models described for adsorption processes. The results show that there is more than one rate-controlling step in the removal process, with diffusion through the boundary layer at the feed/membrane interface being the main rate-controlling step in the removal of 4NA from aqueous solutions by type 1-facilitated transport.

## Data Availability

The data presented in this study are available on request from the corresponding author. The data are not publicly available due to the fact that they are part of a much larger study that is still underway.
